# 

**Published:** 1881-04

**Authors:** 


### Table of Contents,

ORIGINAL COMMUNICATIONS.

Inaugural Thesis for Graduation in the Atlanta Medical College. By D. W. Graham, McVille, Ga........ 1

REPORTS OF SOCIETIES.

Philadelphia Academy of Surgery........ 7
Cincinnati Academy of Medicine........ 15

SELECTIONS.

Clinical Lecture on Arsenical Paralysis........ 31
Pododynia: Its Causes and Significance........ 42
Obliteration of the Lachrymal Passages as a means of Relief from Stricture........ 50
The Fungus of Syphilis........ 51

EDITORIAL.

Vaccination in the Public Schools........ 53
Meteorological Report for March........ 56

BIBLIOGRAPHICAL

The Hygiene and Treatment of Catarrh........ 54
Clinical Lectures on the Physiology, Pathology and Treatment of Syphilis........ 55
Cyclopædia of the Practice of Medicine........ 55

EXCERPTA.

Statistics of Mammary Cancer........ 57
The Laryngeal Obtunder of Krishaber........ 57
The Epidemic of Ergotism in Russia........ 59
A Place where Diplomas are not Needed........ 60
The Abortion of Smallpox—An Important Secret........ 60
Premature Labor Induced by Quinine........ 61
The Wywodzof Method of Preserving Subjects,........ 62
The Treatment of Orchitis and Epididymitis........ 63
Differential Diagnosis of Gastric Ulcer and Cancer........ 63
Longevity of Women........ 64
Simple Continued Fever........ 64
Treatment of Barber's Itch........ 64

